# Effect of antifungal stewardship on micafungin prescribing practices in intensive care units at a tertiary-care hospital

**DOI:** 10.1017/ash.2023.259

**Published:** 2023-09-29

**Authors:** Radhika Arya, Sarah Norman, Farah Daas, Sheena Ramdeen

## Abstract

**Background:** Fungal diseases are associated with substantial global mortality and economic burden, especially in critically ill or immunocompromised patients. Antifungal resistance has emerged as a barrier to treating invasive fungal infections, but antifungal stewardship is still a developing effort due to limited data. Here, we describe the antifungal prescribing practices and the impact of antifungal stewardship on micafungin days of therapy (DOTs) in critical care units. **Methods:** This retrospective study included patients who were admitted to the intensive care unit (ICU) at a tertiary-care hospital in Washington, DC. The preintervention group included baseline micafungin use data between January 1, 2021, and May 31, 2021. The postintervention group included prospective audits, feedback on micafungin orders by a clinical pharmacist, and education on the appropriateness of the antifungal agents. The postintervention group included patients admitted between June 1, 2021, and December 31, 2021. Approval was obtained from the institutional review board. **Results:** The overall average of micafungin days of therapy (DOT) per 1,000 patient days present in the preintervention group versus the postintervention group was 33 versus 24 days, respectively. Moreover, 121 patients were randomly selected for a more detailed retrospective review to define micafungin prescribing practices further. Of these, 73 patients (60.3%) were male; the median age was 63 years. The most common cause for prescribing micafungin in both groups was empiric antifungal coverage (62.8%), followed by fungemia (12.4%). The most common organism isolated was *Candida albicans*. For other sources of infection and organisms isolated, refer to Table 1. In-hospital mortality occurred in 63 (52.06%) patients in both groups. **Conclusions:** Antifungal stewardship through prospective audit and feedback and education by clinical pharmacists decreased micafungin DOTs in critical care units. Empiric prescribing of micafungin is highly prevalent in the ICU despite the low incidence of invasive fungal infections. Although periodic drug utilization reviews and pharmaceutical surveillance can help reduce the prolonged duration of micafungin therapy in the ICU, more robust and routine antifungal stewardship is key to the appropriate use of micafungin to avoid the emergence of antifungal resistance.

**Figure:**
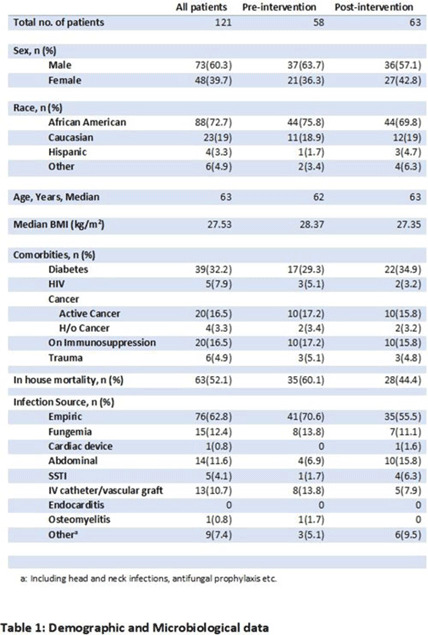

**Figure:**
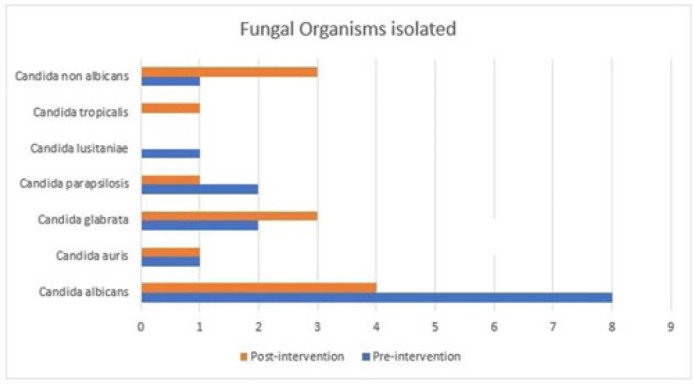

**Disclosures:** None

